# Clinical and Economic Burden of Metabolic Dysfunction-Associated Steatotic Liver Disease (MASLD) in a Spanish Mediterranean Region: A Population-Based Study

**DOI:** 10.3390/jcm14072441

**Published:** 2025-04-03

**Authors:** Javier Díaz Carnicero, Inma Saurí-Ferrer, Josep Redon, Jorge Navarro, Gonzalo Fernández, Carlos Hurtado, Karine Ferreira, Carolina Alvarez-Ortega, Antón Gómez, Carlos J. Martos-Rodríguez, David Martí-Aguado, Desamparados Escudero, Marta Cedenilla

**Affiliations:** 1 Instituto de Investigación Sanitaria Fundación para la Investigación del Hospital Clínico de la Comunidad Valenciana (INCLIVA), Hospital Clínico Universitario, 46010 Valencia, Spain; jdiaz@incliva.es (J.D.C.); isauri@incliva.es (I.S.-F.); josep.redon@uv.es (J.R.); jorgenavper@gmail.com (J.N.); davidmmaa@gmail.com (D.M.-A.); 2 Value & Implementation, Global Medical & Scientific Affairs, MSD Spain, 28027 Madrid, Spain; 3 Gastroenterology and Hepatology, Hospital Clínico Universitario de Valencia, 46010 Valencia, Spain; m.desamparados.escudero@uv.es; 4 Departamento de Medicina, Universidad de Valencia, 46010 Valencia, Spain

**Keywords:** MASLD, MASH, prevalence, complications, costs

## Abstract

**Background/Objectives:** Metabolic dysfunction-associated steatotic liver disease (MASLD) is a prevalent condition worldwide, with significant regional variability in prevalence estimates. This study aimed to determine the prevalence, demographic characteristics, and economic burden of MASLD, metabolic dysfunction-associated steatotic liver (MASL), and metabolic dysfunction-associated steatohepatitis (MASH) in the Valencian Community region of Spain. **Methods:** We conducted a retrospective analysis of electronic medical records from the Valencian public healthcare database of individuals aged over 24 years from 2012 to 2019. **Results:** Of the 3,411,069 individuals included in the database in 2019, 75,565 were diagnosed with MASLD, 74,065 with MASL, and 1504 with MASH based on the International Classification of Diseases (ICD), corresponding to a prevalence of 2.22%, 2.17%, and 0.04%, respectively. Among individuals with type 2 diabetes mellitus (T2DM) or obesity, the prevalence of MASLD was approximately three times and 2.5 times higher, respectively, compared to the overall population. The prevalence of MASLD, MASL, and MASH increased from 2012 to 2019 in all the populations studied. The highest risk of hospitalization was associated with liver-related causes, followed by all-cause hospitalization. The highest cost per subject in 2019 was observed in individuals with concomitant MASH and T2DM. **Conclusions:** Our findings indicate a rising prevalence of MASLD, MASL, and MASH, despite their potential underdiagnosis during the study period. The presence of MASLD or MASH was associated with high healthcare costs, particularly in patients with MASH and T2DM. Our results underline the need for more effective strategies to enhance disease awareness and improve resource allocation.

## 1. Introduction

Metabolic dysfunction-associated steatotic liver disease (MASLD), previously termed non-alcoholic fatty liver disease (NAFLD), is the most prevalent chronic liver disease globally [1,2]. This condition is characterized by the accumulation of fat in the liver with any other cardiometabolic risk factor in the absence of other causes of hepatic steatosis [2,3].

MASLD comprises a broad spectrum of liver conditions, ranging from simple steatosis, known as metabolic dysfunction-associated steatotic liver (MASL), to the more severe metabolic dysfunction-associated steatohepatitis (MASH). MASL is characterized by fat accumulation without significant inflammation or fibrosis, whereas MASH involves steatosis along with inflammation and hepatocyte ballooning [4]. MASH represents the most severe condition, as it can progress to advanced liver fibrosis, cirrhosis, and hepatocellular carcinoma, and it has become the fastest-rising cause of liver transplantation in Western countries [5]. The natural history of MASLD is more complex than initially understood, with the potential for disease progression, regression, or stability. Therefore, identifying patients with advanced steatohepatitis or fibrosis is critical for preventing progression to advanced liver disease and its complications, such as decompensated cirrhosis [6].

A systematic review and meta-analysis recently estimated a worldwide 32% prevalence of MASLD and 5.3% of MASH [7,8]. Epidemiological studies have shown a rising prevalence of MASLD from 25.3% in 1990–2006 to 38.0% in 2016–2019 [8] in parallel with the increasing epidemic of obesity and type 2 diabetes mellitus (T2DM) [9]. However, this trend shows considerable geographic variation influenced by demographic, socio-economic, and genetic differences across regions [10,11]. This variability also arises from the challenges in determining MASLD prevalence because most patients are asymptomatic, difficult to diagnose, and disease awareness remains low [12,13]. In this context, while liver biopsy has traditionally been the gold standard for diagnosing MASH and liver fibrosis, its associated complications and sampling variability have led to the increasing use of non-invasive methods like the fibrosis-4 (FIB-4) index and the NAFLD fibrosis score (NFS) [14,15,16]. FIB-4 and NFS are validated scores to detect the risk of liver fibrosis, which may help identify patients who require further testing and referral [17].

MASLD is increasingly recognized as a multisystem disease associated with hepatic and extrahepatic complications [18]. Insulin resistance and metabolic dysfunction are key drivers of disease progression, not only leading to severe liver-related outcomes such as cirrhosis, liver failure, and hepatocellular carcinoma but also contributing to a broad range of extrahepatic conditions, including cardiovascular disease, chronic kidney disease, T2DM, portal hypertension, ascites, hepatic encephalopathy, and certain types of cancer [19]. MASLD has a bidirectional relationship with metabolic syndrome and is a significant risk factor for major adverse cardiovascular events, being the leading cause of mortality in this population [10]. These comorbidities exacerbate liver fibrosis progression, worsen prognosis, and severely impact the quality of life of patients, thus posing substantial challenges for both patients and healthcare systems [20,21].

Given the increasing prevalence of MASLD coupled with its low disease awareness, understanding the epidemiology of this condition and its economic burden is crucial for identifying areas of improvement and optimizing resource allocation. The aim of this study was to determine the prevalence and economic burden of MASLD in Spain based on the public healthcare database of the Valencian Community region. We observed a rising prevalence of MASLD, MASL, and MASH, despite a potential underdiagnosis of these conditions in clinical practice.

## 2. Materials and Methods

### 2.1. Study Design

This retrospective study was conducted in accordance with the Declaration of Helsinki after approval by the Ethics Committee of Hospital Clínico Universitario de Valencia (protocol code: 2022/174; 27 May 2022). Patient consent was waived due to its observational and retrospective design and because patients were not interviewed at any time during the study.

We analyzed data from the Valencian public healthcare database, which contains electronic medical records for all individuals residing in this region of Spain. The database integrates demographic, socio-economic, and clinical healthcare information across various levels of care, including primary care (ABUCASIS database), hospital care (ORION database), pharmacy, and laboratory data. This study analyzed longitudinal data from 1 January 2012 to 31 December 2019.

### 2.2. Study Population

Data from adults registered in the Valencian public healthcare database aged 24 years or older during the study period were analyzed. We excluded data from individuals who lacked essential information in the database, diagnosed with viral hepatitis, alcoholic hepatitis, toxic hepatitis, and autoimmune hepatitis, or with gestational diabetes mellitus or any secondary diabetes mellitus. Study data included demographic, socio-economic, and clinical information from each patient, regardless of the level of care.

### 2.3. Study Outcomes

The main objective of this study was to determine the clinical and economic burden of MASLD, MASL, and MASH in the Valencian Community region of Spain based on a population database. The primary endpoint of this study was the prevalence of MASLD, MASL, and MASH in the overall population and individuals with T2DM or obesity using each of the following diagnostic criteria: (1) liver biopsy, (2) International Classification of Diseases (ICD)-ninth, tenth revision (ICD-9,10) codes, and (3) the criteria established in the European NAFLD registry and TARGET-NASH study [22,23].

The secondary objectives of this study were: (1) to describe the demographic and clinical characteristics of patients with MASLD, MASL, and MASH; (2) to determine the temporal trends in the prevalence of MASLD, MASL, and MASH between 2012 and 2019; (3) to estimate the risks of clinical complications among patients with MASLD and MASH; and (4) to estimate resource use and its associated costs among patients with MASLD, MASL, and MASH. Analyses were performed in the overall population and individuals with T2DM or obesity. Data on prevalence, demographic characteristics, clinical complications, healthcare use, and costs are presented relative to 2019. Since the study was designed and conducted before the change in NAFLD/MASLD terminology, ICD-9,10 codes for NAFL and NASH were used. The prevalence of NAFLD was calculated by summing the prevalence rates of NAFL and NASH based on these ICD-9,10 codes.

To estimate associated risks, the following complications were considered: hospitalizations (comprising all-cause, cardiovascular-, renal- and liver-related causes) and mortality. Liver-related causes included cirrhosis and its associated complications (ascites, variceal bleedings, encephalopathy, or worsening of MELD score [especially ≥ 15]), hepatocellular carcinoma, and liver transplantation. Mortality included all causes and in-hospital mortality due to cardiovascular-, renal-, and liver-related causes. The diagnosis-related groups (DRGs) used for hospitalizations due to cardiovascular-, renal-, and liver-related causes were obtained from the Spanish Ministry of Health [24]. To assess healthcare resource utilization, the following variables were collected: primary care visits, pharmacy electronic prescriptions, hospital outpatient visits, major ambulatory surgery, hospitalizations, and emergency visits.

Healthcare costs were estimated using standard costs, as financial billing information by patients is not available in public healthcare centers. The primary sources for these standard costs were the Rate Law and the System Economic Information [25]. Additionally, pharmaceutical expenditure was obtained from the e-prescription system.

### 2.4. Statistical Analysis and Sample Size

All analyses were performed using the R software (version 6.3.1). Prevalences of MASLD, MASL, and MASH were calculated based on the accumulated prevalence at the end of each year during the study period. Data from individuals diagnosed with MASLD, MASL, and MASH between 1 January 2012 and 31 December 2019 were considered. The proportion of people who progressed from MASL to MASH during the study period was also calculated. A Cox proportional hazards model was used to estimate the hazard ratios (HRs) for the relative risks of complications (hospitalizations and mortality).

## 3. Results

### 3.1. Study Population

The database comprised 3,743,302 individuals in 2012 and 3,411,069 in 2019. Data from 126,902 individuals were excluded from the analysis throughout the study period and from 4143 individuals in 2019. Of the 3,411,069 individuals included in 2019, 75,565 were diagnosed with MASLD, 74,065 with MASL, and 1504 with MASH based on ICD codification. The mean age in the overall population was 61.92 years. Patients with MASH had higher body mass index (BMI), fasting glucose, triglycerides, aspartate aminotransferase (AST), and alanine aminotransferase (ALT) levels compared to those with MASLD and MASL (Table 1). The T2DM population comprised 405,918 individuals (11.90%), with 27,701 diagnosed with MASLD, 27,056 with MASL, and 645 with MASH. The obesity population included 693,653 individuals (20.34%): 39,346 were diagnosed with MASLD, 46,235 with MASL, and 779 with MASH (Supplementary Table S1). Demographic characteristics were similar within the T2DM and obesity subpopulations to those observed in the overall population (Supplementary Table S1).

### 3.2. Prevalence

**Figure 1:**
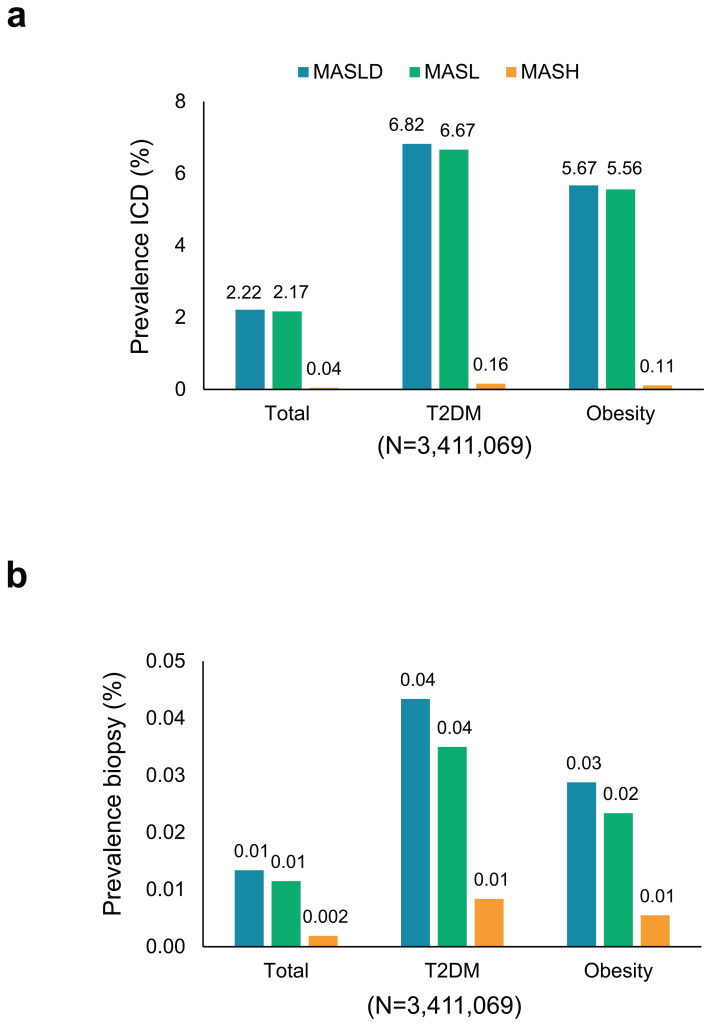
MASLD, MASL, and MASH prevalence in the overall population and in individuals with type 2 diabetes mellitus or obesity. The graphs show the proportion of individuals with MASLD, MASL, and MASH based on (**a**) ICD codification and (**b**) liver biopsy. ICD, International Classification of Diseases; MASLD, metabolic dysfunction-associated steatotic liver disease; MASL, metabolic dysfunction-associated steatotic liver; MASH, metabolic dysfunction-associated steatohepatitis; T2DM, type 2 diabetes mellitus.

The 2019 prevalence of MASLD, MASL, and MASH based on ICD codification, was 2.22%, 2.17%, and 0.04%, respectively (Figure 1a). Among individuals with T2DM or obesity, these prevalences were approximately three times (6.82%) and 2.5 times (5.67%) higher, respectively, compared to the overall population (Figure 1a). Notably, MASH prevalence was four times higher in the T2DM population compared to the overall population. Prevalences determined by biopsy were lower but followed a similar pattern across subpopulations (Figure 1b). Due to the lack of histology or radiology results in the database, none of the European NAFLD registry criteria could be fulfilled. Likewise, due to insufficient data in the database for appropriate calculation, it was not possible to apply the criteria used in the TARGET-NASH registry. Subsequent results are therefore presented using ICD-9,10 codes only.

The prevalence of MASLD and MASL was highest among individuals aged 55–64 years and declined with increasing age (Supplementary Figure S1). MASH prevalence peaked in the 55–64 age group in the overall population and among individuals with obesity, while it was highest in those under 55 years old among people with T2DM (Supplementary Figure S1). A higher prevalence of these conditions was observed among men than women in the overall population and in individuals with obesity. In contrast, the prevalence of the three conditions was lower in men versus women with T2DM (Supplementary Figure S2).

**Figure 2:**
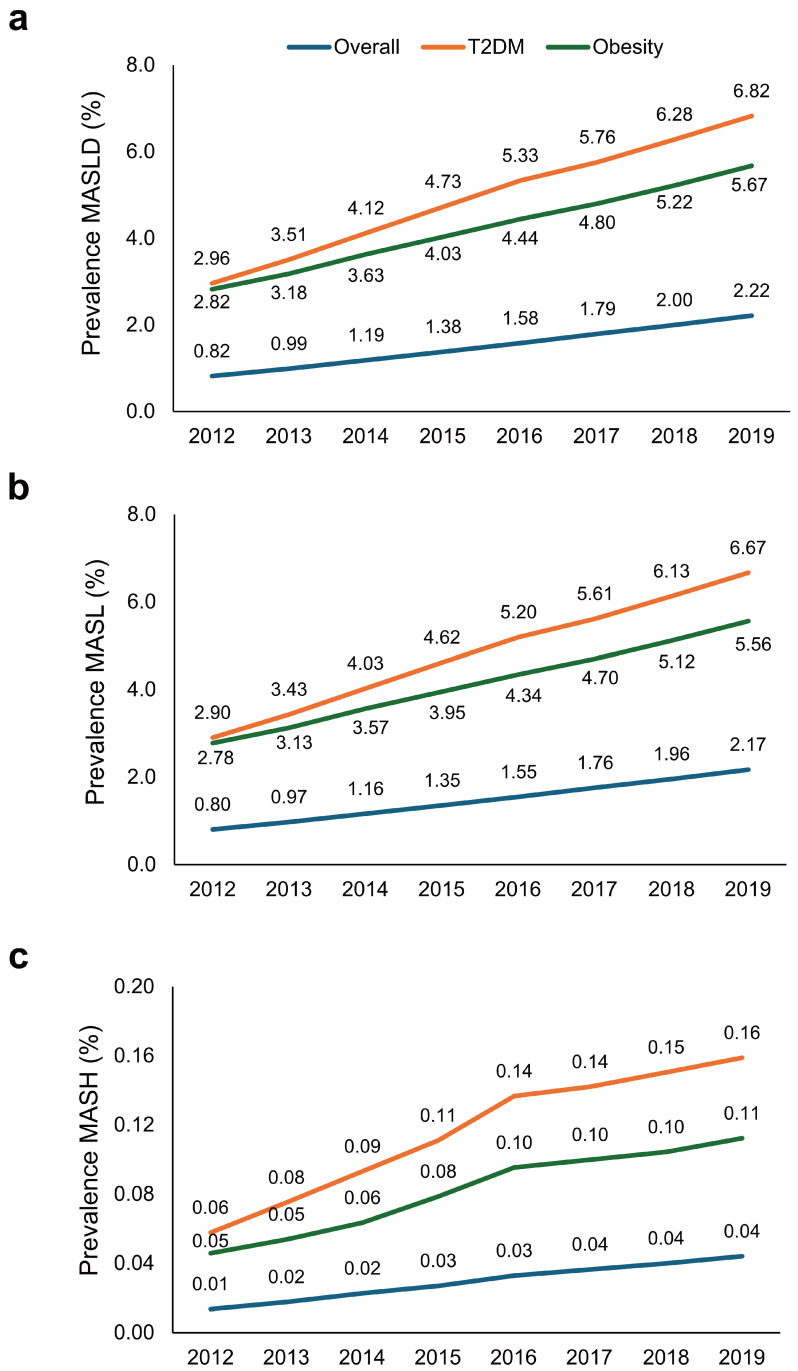
Trends in MASLD, MASL, and MASH prevalence in the overall population and in individuals with type 2 diabetes mellitus or obesity. The graphs show the prevalence of (**a**) MASLD, (**b**) MASL, and (**c**) MASH from 2012 to 2019. MASLD, metabolic dysfunction-associated steatotic liver disease; MASL, metabolic dysfunction-associated steatotic liver; MASH, metabolic dysfunction-associated steatohepatitis; T2DM, type 2 diabetes mellitus.

The prevalence of MASLD, MASL, and MASH steadily increased from 2012 to 2019 in the overall population and in individuals with T2DM or obesity (Figure 2).

### 3.3. Complications and Resource Use

**Figure 3:**
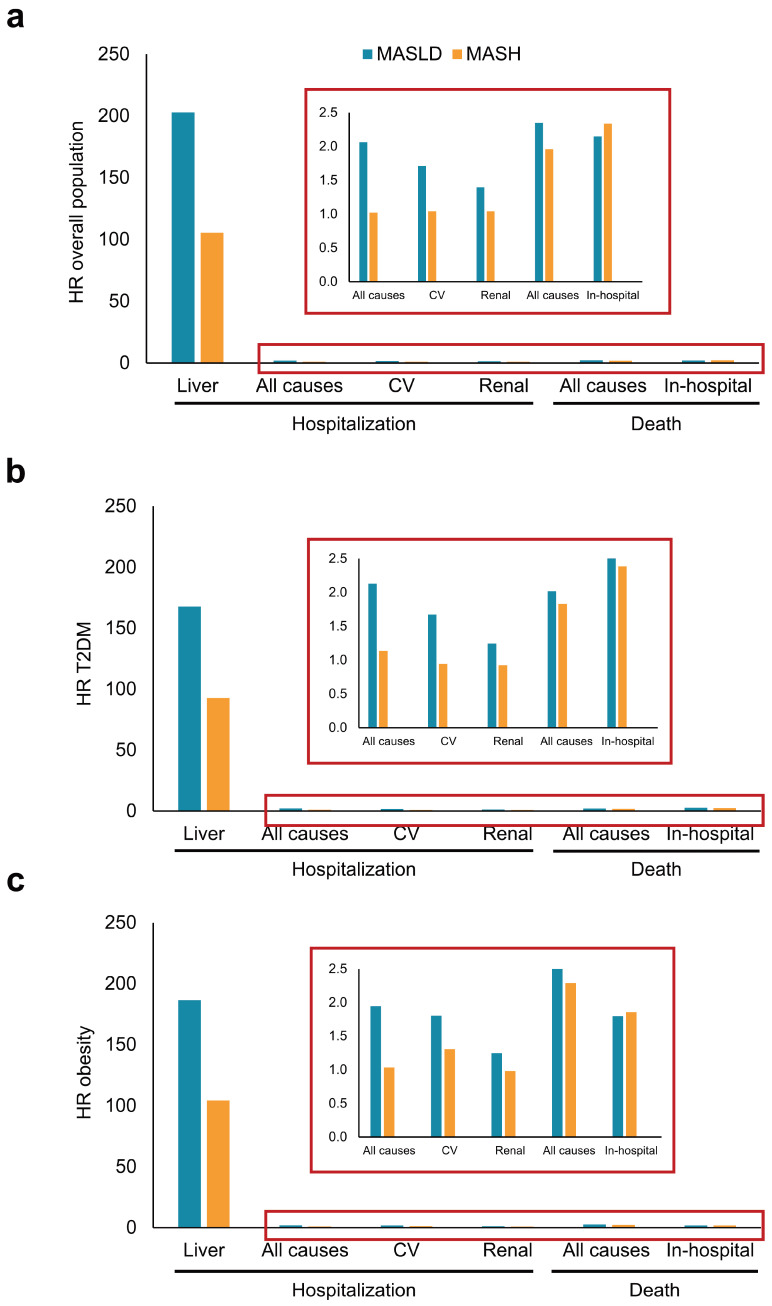
Annual relative risk of complications among patients with MASLD and MASH. The bar graphs show Hazard Ratios (HR) among individuals with MASLD and MASH in (**a**) the overall population and the populations with (**b**) type 2 diabetes mellitus (T2DM) and (**c**) obesity. CV, cardiovascular; MASLD, metabolic dysfunction-associated steatotic liver disease; MASH, metabolic dysfunction-associated steatohepatitis.

Regarding complications, the highest risk of hospitalization was due to liver-related causes across all populations. In the overall population, patients with MASLD and MASH showed an increased risk of both all-cause (HR: 2.35 [95% CI, 1.98–2.79] and 1.96 [95% CI, 1.73–2.22], respectively) and in-hospital death (HR: 2.15 [95% CI, 1.03–4.51] and 2.34 [95% CI, 1.45–3.76], respectively). Individuals with T2DM and MASLD or MASH also exhibited an elevated risk of in-hospital death (HR: 2.66 [95% CI, 1.27–5.58] and 2.39 [95% CI, 1.41–4.0], respectively). Excluding liver-related causes, the highest HR was found for all-cause death among individuals with MASLD (HR: 2.75 [95% CI, 2.15–3.51]) or MASH (HR: 2.29 [95% CI, 1.92–2.73]) in the population with obesity (Figure 3).

**Figure 4:**
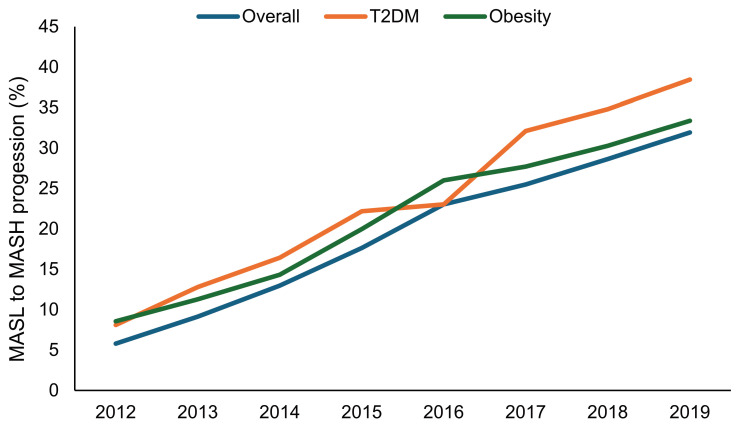
Proportion of patients who progressed from MASL to MASH in the overall population and in individuals with type 2 diabetes mellitus or obesity from 2012 to 2019. MASL, metabolic dysfunction-associated steatotic liver; MASH, metabolic dysfunction-associated steatohepatitis; T2DM, type 2 diabetes mellitus.

The proportion of people at risk of progression from MASL to MASH increased in the overall population and in individuals with T2DM and obesity from 2012 to 2019 (Figure 4).

Most individuals with MASLD, MASL, or MASH attended primary care visits, hospital outpatient visits, or used pharmacy electronic prescriptions at least once in 2019, with the highest use in the T2DM population across all conditions. Annual hospitalizations and emergency visits were more common among patients with concomitant MASH and T2DM, for whom the rate of emergency visits reached 40.78% (Table 2). Higher annual costs per subject were observed in individuals with MASH compared to MASL and MASLD across overall, T2DM, and obesity populations. The highest annual costs per subject were associated with individuals with both MASH and T2DM (Table 2).

## 4. Discussion

In this study, we determined the prevalence, demographic characteristics, and economic burden of MASLD, MASL, and MASH in the overall population and in individuals with T2DM and obesity within the Valencian Community region of Spain. Our findings reveal a steadily increasing prevalence of these conditions over a seven-year period, with higher prevalence rates among individuals with T2DM or obesity. Our results suggest underdiagnosis of these conditions in clinical practice, highlighting a clinical unmet need for screening, diagnosis, and optimal resource allocation.

The increasing prevalence of MASLD/MASH emphasizes the need for early diagnosis, which is crucial for effective risk assessment and preventing disease progression and complications [3,26]. In this context, the prevalence rates observed in our study—2.22% and 2.17% for MASLD and MASL, respectively, and 0.04% for MASH—are notably lower than global estimates. For instance, a 2016 meta-analysis estimated a global prevalence of MASLD at 25.2% and another conducted in 2022 at 32% [7,9]. In Spain, only a few studies have assessed the prevalence of these conditions [27,28,29]. One such study conducted in 2012 using ultrasound criteria estimated a MASLD prevalence of 25.8% [28,29]. Discrepancies between our data and those previously published may be associated with several factors, including differences in study design, studied population, diagnostic methods, and case definitions [10,20]. While standardized, the use of ICD codification may not capture all cases of MASLD, particularly those in earlier stages or those not actively managed in healthcare settings. In fact, studies using diagnosis codes and liver enzymes often report lower MASLD prevalences due to undercoding and normal enzyme levels. In contrast, though more accurate and yield higher prevalence estimates, those based on MRI-based methods are costly and typically involve smaller sample sizes [10]. Therefore, while regional epidemiological factors may have contributed to differences in prevalence rates, we believe our rates are lower than global estimates primarily due to the use of ICD codification, which leads to underestimation. Although this may appear to be a limitation of this study, it also highlights that this condition is not widely recognized by physicians. Notably, the prevalence of MASLD in the general population of the Valencian Community region was previously estimated at 34%, aligning with recent estimates [30].

This underdiagnosis could arise from a lack of awareness among healthcare providers and patients and a limited use of diagnostic tools. Specifically, a study showed that MASLD is significantly underdiagnosed in primary care, often due to a lack of prioritization and low awareness among healthcare providers [12]. This is reflected in multinational and in studies from the USA in primary care, which show a MASLD prevalence far below estimated population rates [31]. Similarly, an observational study conducted in Spain estimated a prevalence of 2.17% for hepatic steatosis and 1.51% for MASLD among all patients who attended primary care in an urban healthcare center [32], which aligns more closely with our results. This idea is further supported by the increasing trend in prevalence observed over time, which may reflect a combination of rising prevalence and improved detection. In recent years, non-invasive tests and biomarkers, imaging techniques, and scoring panels have contributed to improving the diagnosis of MASLD. As a result, a combination of non-invasive methods is increasingly used in clinical practice to identify and stage liver conditions [26].

The higher prevalence of MASLD, MASL, and MASH among individuals with T2DM and obesity aligns with the well-established link between metabolic syndrome and liver disease. T2DM and obesity are major risk factors for the development and progression of MASLD, significantly increasing the risk of advanced liver disease, including MASH and cirrhosis. Our data show that individuals with T2DM or obesity have a two- to three-fold higher prevalence of MASLD compared to the overall population, and four times higher for MASH in the T2DM population compared to the overall population. In a recent study, the prevalence of MASLD was 67.3% among individuals with obesity or overweight [33]. A prospective study showed that the prevalence of MASLD, advanced fibrosis, and cirrhosis in adults aged 50 years or older with T2DM was 65%, 14%, and 6%, respectively [34]. Furthermore, a cross-sectional analysis of the 2017–2018 National Health and Nutrition Examination Survey in the USA showed that subjects with comorbidities such as diabetes, obesity, metabolic syndrome, and insulin resistance had a 1.3 to 1.7 times higher prevalence of MASH compared to the general population [35]. Notably, this study from the USA also showed that patients with MASLD and T2DM had the highest prevalence of at-risk MASH [35]. Similarly, in a cross-sectional study conducted in Spain, the prevalence of MASLD among patients with metabolic syndrome was 43% [28]. In another Spanish observational study, 50% of patients with obesity and T2DM exhibited MASLD-associated intense steatosis, while 20% showed MASLD-associated fibrosis [36]. These results underscore the importance of early screening of patients with T2DM or obesity for metabolic liver disease.

The increased risk of liver-related and all-cause hospitalizations and mortality in MASLD and MASH populations indicates that the clinical impact of these conditions extends beyond liver-specific complications. In a study modeling MASLD disease burden in eight countries (China, Germany, France, Italy, Japan, Spain, the UK, and the USA), Spain showed the lowest prevalence of liver-related deaths [37]. Additionally, a recent systematic review and meta-analysis of population-based studies from 1990 to 2019 found that, within the MASLD population, the pooled mortality rate was 12.6 per 1000 person-years for all-cause mortality, 4.2 per 1000 person-years for cardiac-specific mortality, 2.8 per 1000 person-years for extrahepatic cancer-specific mortality, and 0.92 per 1000 person-years for liver-specific mortality [8].

The high risk of complications observed aligns with the significant economic burden associated with MASLD, particularly for patients with MASH and concomitant complications. Individuals with MASH were associated with higher healthcare costs compared to those with MASLD and MASL. This finding is consistent with the fact that MASH represents a more severe form of liver disease, leading to increased morbidity and mortality. Indeed, the highest cost was associated with patients with concomitant MASH and T2DM, in whom the annual total cost per subject was €4295.65. Previous studies evaluating the economic burden found that total costs are higher in the USA compared to Europe, with increases observed over time and across disease severity stages [38]. In a retrospective, cross-sectional study across Europe (France, Italy, Germany, Spain, and the UK) and the USA, the mean annual total MASH-related costs in Spain were €2162 [39], slightly lower than that found in the MASH population of our study (€3203.91). These findings highlight the importance of early detection and intervention of MASLD, particularly in patients with T2DM and obesity, to prevent progression to more severe disease and reduce the associated healthcare costs.

Our findings emphasize the need to implement strategies that improve disease awareness and optimize resource allocation. Such strategies may include establishing early screening programs, refining diagnostic criteria, and integrating risk-based stratification methods. However, low disease awareness and underdiagnosis remain important barriers to prevention, early detection, and efficient MASLD management, including lifestyle modifications and pharmacotherapy [40]. To facilitate early detection and intervention, the EASL–EASD–EASO Clinical Practice Guidelines recommend that healthcare providers look for MASLD with liver fibrosis in individuals with T2DM, abdominal obesity with at least one additional metabolic risk factor, or abnormal liver function tests [16].

Recent evidence supports the cost-effectiveness of screening for high-risk MASLD [41], while another study found that sequential non-invasive test screening strategies are both cost-saving and efficient for managing high-risk MASLD patients [42].

This study has several limitations that should be acknowledged. The use of a retrospective public database may have underestimated MASLD prevalences due to potential undercoding or misclassification of diagnoses. Additionally, the low prevalence of certain conditions, such as biopsy-confirmed or cases documented in the TARGET-NASH registry, limited the ability to perform analyses in these subgroups. Furthermore, the results are specific to the Valencian Community region of Spain and may not be generalizable to other populations. Finally, indirect costs, such as loss of productivity, disability-adjusted life years, or caregiver burden, were not considered in our analysis, which could have underestimated the overall economic burden associated with these conditions.

## 5. Conclusions

This is the first study addressing the clinical and economic burden of MASLD, MASL, and MASH from a large Spanish database. Our findings reveal an increasing prevalence of MASLD, MASL, and MASH, although they seem to be underdiagnosed throughout the study period. Furthermore, these conditions were associated with an increased risk of complications and use of healthcare resources, leading to a significant economic burden, particularly for individuals with T2DM and MASH. These results emphasize the need for more effective strategies to raise disease awareness, improve screening and diagnosis in at-risk populations, and optimize resource allocation.

## Figures and Tables

**Table 1:** Demographic and clinical characteristics of individuals with MASLD, MASL, and MASH.

Overall Population (N = 3,411,069)			
Variables, Mean (SD)	MASLD (N = 75,565)	MASL (N = 74,065)	MASH (N = 1504)
Age (years)	61.93 (12.51)	61.94 (12.52)	61.89 (11.91)
Sex			
Men, n (%)	41,158 (54.47%)	40,652 (54.89%)	745 (49.57%)
Weight (kg)	84.21 (16.92)	84.22 (16.93)	83.36 (16.03)
BMI (kg/m^2^)	31.84 (5.54)	31.83 (5.54)	32.01 (5.61)
Waist circumference (cm)	107.46 (11.2)	107.47 (11.22)	107.90 (10.67)
SBP (mm Hg)	131.36 (13.15)	131.36 (13.16)	131.31 (13.58)
DBP (mm Hg)	78.38 (9.54)	78.39 (9.54)	77.70 (9.57)
Diabetes duration (years)	7.44 (3.83)	7.43 (3.84)	8.12 (3.90)
HbA_1c_ (%)	6.40 (1.12)	6.41 (1.12)	6.42 (1.14)
Fasting glucose (mg/dL)	112.37 (32.95)	112.36 (32.95)	115.03 (34.91)
Total cholesterol (mg/dL)	187.03 (40.92)	187.02 (40.91)	185.91 (41.66)
HDL cholesterol (mg/dL)	50.88 (13.52)	50.88 (13.52)	51.02 (14.07)
LDL cholesterol (mg/dL)	105.91 (34.55)	105.92 (34.54)	103.67 (34.94)
Triglycerides (mg/dL)	162.70 (96.08)	162.67 (96.12)	169.14 (106.19)
Creatinine (mg/dL)	0.88 (0.38)	0.88 (0.38)	0.90 (0.51)
Albumin (g/L)	26.67 (44.50)	26.55 (44.31)	35.2 (55.90)
eGFR (ml/min/1.73 m^2^)	80.37 (19.68)	80.38 (19.66)	79.07 (21.94)
AST (U/L)	29.51 (18.74)	29.45 (18.76)	33.51 (18.54)
ALT (U/L)	31.37 (22.65)	31.32 (22.65)	34.9 (22.73)
Platelets (×10^9^/L)	237.31 (66.89)	237.42 (66.82)	225.2 (73.45)
Uric acid (mg/dL)	5.69 (1.51)	5.69 (1.51)	5.62 (1.51)
Iron (μg/dL)	84.96 (32.32)	84.94 (32.32)	87.72 (32.77)
Ferritin (ng/mL)	154.12 (152.77)	154.37 (152.78)	139.38 (147.43)
Transferrin (mg/dL)	270.19 (52.14)	270.21 (52.23)	270.29 (47.27)
Leucocytes (×10⁹/L)	7.36 (2.04)	7.37 (2.04)	7 (2.03)
CRP (nmol/L)	6.24 (15.55)	6.26 (15.64)	4.64 (7.36)
Vitamin D (ng/mL)	25.39 (12.81)	25.39 (12.81)	27.24 (15.38)

Data are expressed as mean (standard deviation, SD) unless otherwise specified. ALT, alanine aminotransferase; AST, aspartate aminotransferase; BMI, body mass index; CRP, C-reactive protein; DBP, diastolic blood pressure; eGFR, estimated glomerular filtration rate; HbA1c, glycated hemoglobin; HDL, high-density lipoprotein; LDL, low-density lipoprotein; MASLD, metabolic dysfunction-associated steatotic liver disease; MASL, metabolic dysfunction-associated steatotic liver; MASH, metabolic dysfunction-associated steatohepatitis; SBP, systolic blood pressure; SD, standard deviation.

**Table 2:** Annual use of healthcare resources and associated costs in individuals with MASLD, MASL, and MASH in the overall, T2DM, and obesity populations.

	Overall Population			T2DM			Obesity		
Variable	%Use	Total Cost (€)	Cost per Subject (€)	%Use	Total Cost (€)	Cost per Subject (€)	%Use	Total Cost (€)	Cost per Subject (€)
**MASLD**									
Primary care visits	95.86	57,296,977.99	758.23	98.44	25,599,539.84	924.14	97.45	34,093,659.81	866.51
Pharmacy electronic prescription	93.60	72,251,037.57	956.12	97.93	46,173,509.78	1666.85	96.02	47,157,707.98	1198.54
Hospital outpatient visits	76.27	33,240,748.32	439.88	81.50	14,732,332.68	531.83	79.25	19,388,913.44	492.78
Major ambulatory surgery	2.21	2,008,778.38	26.58	2.61	917,795.48	33.13	2.66	1,270,041.08	32.28
Hospitalizations	5.83	28,884,119.34	382.23	7.13	13,666,333.30	493.35	6.95	18,397,447.16	467.58
Emergency visits	34.85	9,792,274.73	129.58	38.99	4,221,079.24	152.38	37.73	5,688,489.80	144.58
Total Cost (€)		203,473,936.33	2692.63		105,310,590.32	3801.69		125,996,259.27	3202.26
**MASL**									
Primary care visits	95.85	56,565,946.29	758.83	98.43	25,259,074.00	925.11	97.43	33,651,314.19	866.70
Pharmacy electronic prescription	93.60	71,187,372.89	954.97	97.91	45,462,457.55	1665.05	96.01	46,477,787.07	1197.05
Hospital outpatient visits	76.19	32,622,010.20	437.62	81.43	14,459,701.36	529.58	79.14	19,052,837.96	490.71
Major ambulatory surgery	2.21	1,982,359.96	26.59	2.61	905,075.50	33.15	2.66	1,253,407.26	32.28
Hospitalizations	5.82	28,401,510.54	381.00	7.12	13,458,034.47	492.90	6.94	18,078,086.38	465.61
Emergency visits	34.84	9,657,357.85	129.55	38.99	4,160,063.46	152.36	37.73	5,614,967.68	144.62
Total Cost (€)		200,416,557.73	2688.57		103,704,406.34	3798.14		124,128,400.54	3196.96
**MASH**									
Primary care visits	96.61	1,124,809.46	748.38	99.07	572,832.03	888.11	98.46	691,903.98	888.20
Pharmacy electronic prescription	94.68	1,749,865.26	1164.25	98.91	1,250,765.59	1939.17	97.30	1,160,195.36	1489.34
Hospital outpatient visits	84.96	990,632.24	659.10	88.53	507,920.92	787.47	88.70	574,358.72	737.30
Major ambulatory surgery	2.33	44,030.70	29.30	2.79	23,483.04	36.41	2.82	27,396.88	35.17
Hospitalizations	6.45	679,325.61	451.98	6.67	305,220.33	473.21	7.96	482,230.36	619.04
Emergency visits	37.79	226,819.53	150.91	40.78	110,472.67	171.28	39.92	133,969.43	171.98
Total Cost (€)		4,815,482.80	3203.91		2,770,694.58	4295.65		3,070,054.73	3941.02

The table shows the annual proportion of patients who used each resource and its associated costs relative to 2019. MASLD, metabolic dysfunction-associated steatotic liver disease; MASL, metabolic dysfunction-associated steatotic liver; MASH, metabolic dysfunction-associated steatohepatitis; T2DM, type 2 diabetes mellitus.

## Data Availability

The dataset is available on request due to restrictions for privacy reasons.

## Supplementary Materials

The following supporting information can be downloaded at: https://www.mdpi.com/article/10.3390/jcm14072441/s1. Table S1. Demographic and clinical characteristics of individuals with MASLD, MASL, and MASH across type 2 diabetes mellitus and obesity populations; Figure S1. MASLD, MASL, and MASH prevalences across age subgroups in the overall population and in individuals with type 2 diabetes mellitus or obesity. The graphs show the proportion of individuals with (a) MASLD, (b) MASL, and (c) MASH across age subgroups. MASLD, metabolic dysfunction-associated steatotic liver disease; MASL, metabolic dysfunction-associated steatotic liver; MASH, metabolic dysfunction-associated steatohepatitis; T2DM, type 2 diabetes mellitus; Figure S2. MASLD, MASL, and MASH prevalences across men and women in the overall population and in individuals with type 2 diabetes mellitus or obesity. The graphs show the proportion of individuals with (a) MASLD, (b) MASL, and (c) MASH across men and women. MASLD, metabolic dysfunction-associated steatotic liver disease; MASL, metabolic dysfunction-associated steatotic liver; MASH, metabolic dysfunction-associated steatohepatitis; T2DM, type 2 diabetes mellitus.

## Abbreviations

The following abbreviations are used in this manuscript:

MASLD	Metabolic dysfunction-associated steatotic liver disease
MASL	Metabolic dysfunction-associated steatotic liver
MASH	Metabolic dysfunction-associated steatohepatitis
ICD	International Classification of Diseases
T2DM	Type 2 diabetes mellitus
NAFLD	Non-alcoholic fatty liver disease
FIB-4	Fibrosis-4
NFS	NAFLD fibrosis score
DRGs	Diagnosis-related groups
HRs	Hazard ratios
BMI	Body mass index
AST	Aspartate aminotransferase
ALT	Alanine aminotransferase
SD	Standard deviation
CRP	C-reactive protein
DBP	Diastolic blood pressure
eGFR	Estimated glomerular filtration rate
HbA1c	Glycated hemoglobin
HDL	High-density lipoprotein
LDL	Low-density lipoprotein
SBP	Systolic blood pressure
CV	Cardiovascular
