# Supplementary material for: Clinical and Economic Burden of Metabolic Dysfunction-Associated Steatotic Liver Disease (MASLD) in a Spanish Mediterranean Region: A Population-Based Study
Source: J Clin Med. 2025 Apr 3;14(7):2441. doi: 10.3390/jcm14072441 (PMC11989979; doi:10.3390/jcm14072441)
Supplement: Supplementary file 1 [file jcm-14-02441-s001.zip › jcm-3529030-supplementary.pdf]

Supplementary Table S1. Demographic and clinical characteristics of individuals with MASLD, MASL, and MASH across type 2 diabetes mellitus and obesity populations

	T2DM			Obesity		
**Variables, mean (SD)**	**MASLD (N = 75,565)**	**MASL (N = 74,065)**	**MASH (N = 1,504)**	**MASLD (N = 75,565)**	**MASL (N = 74,065)**	**MASH (N = 1,504)**
Age (years)	66.26 (11.1)	66.26 (11.1)	65.56 (10.68)	62.62 (12.45)	62.63 (12.46)	62.8 (11.54)
Sex						
Men, n (%)	14,199 (51.26)	14,024 (51.36)	285 (44.19)	1,931,100 (49.08)	1,909,600 (49.18)	33,000 (42.36)
Weight (kg)	83.91 (16.6)	83.94 (16.61)	82.52 (15.37)	88.48 (16.17)	88.5 (16.17)	87.42 (15.45)
BMI (kg/m^2)	31.96 (5.49)	31.96 (5.49)	32.04 (5.53)	33.57 (5.02)	33.57 (5.02)	33.77 (5.16)
Waist circumference (cm)	107.87 (11.22)	107.88 (11.25)	108.09 (9.8)	110.13 (10.51)	110.16 (10.52)	110.19 (10.45)
SBP (mm Hg)	132.36 (13.3)	132.36 (13.3)	132.28 (14.68)	131.76 (13.11)	131.76 (13.11)	131.14 (13.99)
DBP (mm Hg)	77.55 (9.42)	77.56 (9.42)	77.04 (9.86)	78.46 (9.54)	78.47 (9.53)	77.59 (10.02)
Diabetes duration (years)	7.44 (3.83)	7.43 (3.84)	8.12 (3.88)	7.51 (3.82)	7.51 (3.82)	8.25 (3.95)
HbA$_{1c}$ (%)	6.84 (1.17)	6.84 (1.17)	6.86 (1.17)	6.51 (1.15)	6.51 (1.15)	6.51 (1.21)
Fasting glucose (mg/dL)	132.43 (39.82)	132.43 (39.83)	134.24 (41.53)	116.46 (35.17)	116.43 (35.15)	120.82 (38.74)
Total cholesterol (mg/dL)	175.22 (40.23)	175.24 (40.22)	173.46 (40.65)	183.31 (40.31)	183.31 (40.3)	181.02 (41.08)
HDL cholesterol (mg/dL)	48.1 (12.63)	48.09 (12.62)	48.56 (13.31)	49.58 (12.74)	49.58 (12.74)	49.21 (12.94)
LDL cholesterol (mg/dL)	94.76 (32.77)	94.79 (32.76)	92.12 (33.38)	102.48 (33.84)	102.5 (33.83)	99.42 (34.28)
Triglycerides (mg/dL)	176.6 (105.13)	176.61 (105.18)	184.91 (124.47)	167.81 (97.54)	167.82 (97.63)	174.64 (115.54)
Creatinine (mg/dL)	0.92 (0.45)	0.92 (0.45)	0.97 (0.66)	0.89 (0.41)	0.89 (0.41)	0.93 (0.61)
Albumin (g/L)	32.23 (49.37)	32.1 (49.23)	42.47 (59.71)	28.92 (46.55)	28.76 (46.29)	42.56 (62.56)
eGFR (ml/min/1.73 m^2)	77.23 (20.82)	77.26 (20.81)	75.25 (23.08)	79.62 (20.42)	79.66 (20.4)	76.78 (23)
AST (U/L)	29 (18.51)	28.92 (18.5)	34.63 (19.01)	29.13 (17.86)	29.05 (17.85)	34.26 (18.46)
ALT (U/L)	30.37 (22.19)	30.32 (22.19)	33.68 (21.46)	31.26 (22.05)	31.21 (22.04)	34.95 (22.49)
Platelets (x10^9/L)	234.44 (69.54)	234.7 (69.42)	211.58 (75.65)	236.71 (66.98)	236.8 (66.84)	223.33 (74.88)
Uric acid (mg/dL)	5.57 (1.54)	5.57 (1.54)	5.63 (1.56)	5.74 (1.53)	5.74 (1.53)	5.72 (1.54)
Iron (µg/dL)	77.88 (30.58)	77.88 (30.58)	79.97 (32.13)	81.17 (31.32)	81.14 (31.33)	83.68 (31.87)
Ferritin (ng/mL)	131.63 (143.47)	131.66 (143.24)	127.61 (152.02)	143.33 (147.29)	143.38 (147.08)	129.1 (142.76)
Transferrin (mg/dL)	277.04 (56.54)	277.09 (56.62)	278.01 (53.09)	272.26 (52.92)	272.35 (52.98)	271.26 (51.22)
Leucocytes (× 10^9/L)	7.64 (2.1)	7.65 (2.1)	7.02 (2.19)	7.46 (2.03)	7.46 (2.03)	7.14 (1.97)
CRP (nmol/L)	6.71 (16.53)	6.72 (16.61)	5.31 (8.78)	6.31 (15.11)	6.31 (15.18)	5.67 (8.87)
Vitamin D (ng/mL)	24.81 (12.79)	24.82 (12.79)	25.27 (13.27)	24.39 (12.27)	24.4 (12.28)	24.31 (14.07)

Data are expressed as mean (standard deviation, SD) unless otherwise specified.

ALT, alanine aminotransferase; AST, aspartate aminotransferase; BMI, body mass index; CRP, C-reactive protein; DBP, diastolic blood pressure; eGFR, estimated glomerular filtration rate; HbA$_{1c}$, glycated hemoglobin; HDL, high-density lipoprotein; LDL, low-density lipoprotein; MASLD,

metabolic dysfunction-associated steatotic liver disease; MASL, metabolic dysfunction-associated steatotic liver; MASH, metabolic dysfunction–associated steatohepatitis; T2DM, type 2 diabetes mellitus; SBP, systolic blood pressure; SD, standard deviation.

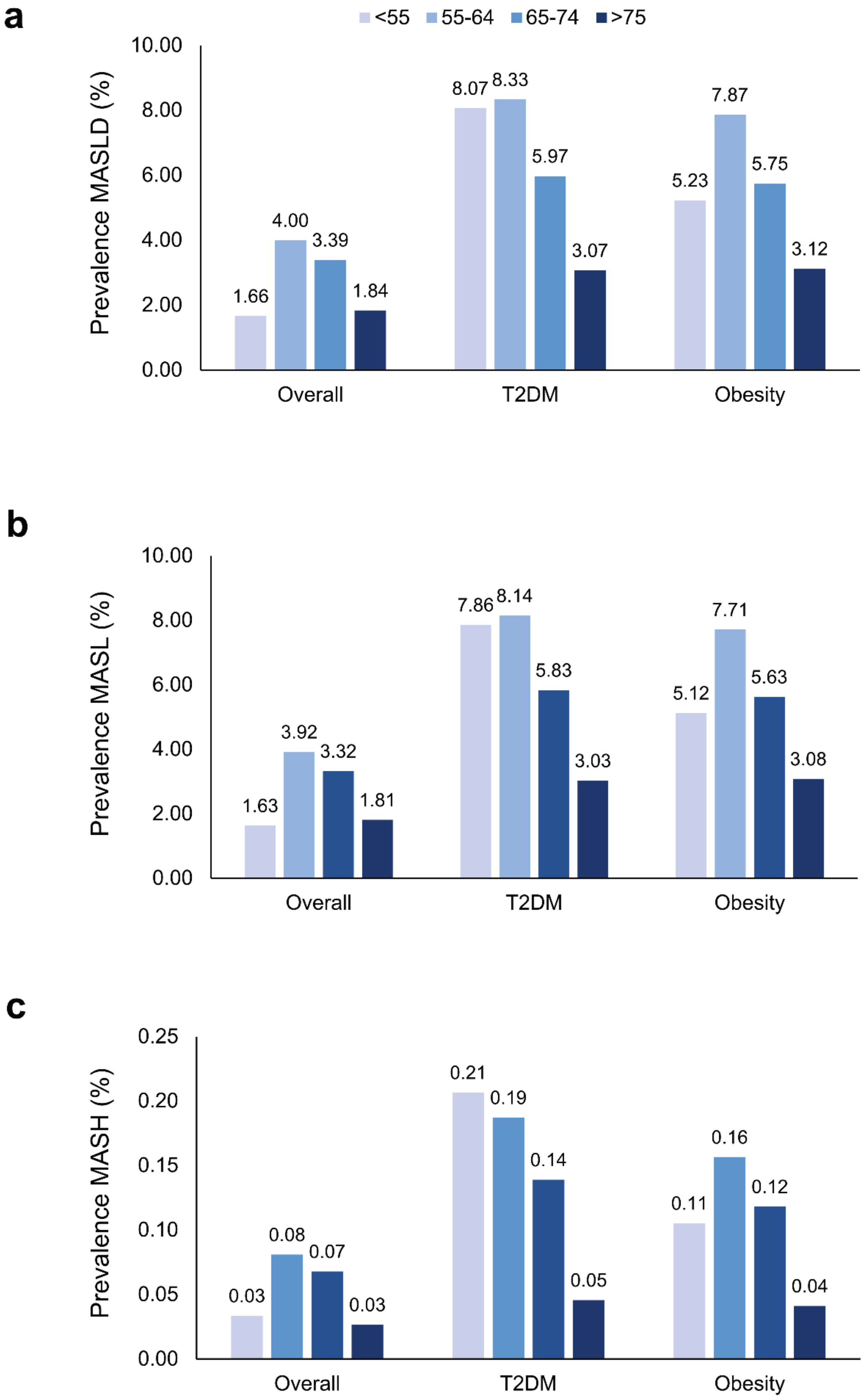
a
<55
55-64
65-74
>75
Prevalence MASLD (%)
10.00
8.00
6.00
4.00
2.00
0.00
1.66
4.00
3.39
1.84
8.07
8.33
5.97
3.07
5.23
7.87
5.75
3.12
Overall
T2DM
Obesity
b
Prevalence MASL (%)
10.00
8.00
6.00
4.00
2.00
0.00
1.63
3.92
3.32
1.81
7.86
8.14
5.83
3.03
5.12
7.71
5.63
3.08
Overall
T2DM
Obesity
c
Prevalence MASH (%)
0.25
0.20
0.15
0.10
0.05
0.00
0.03
0.08
0.07
0.03
0.21
0.19
0.14
0.05
0.11
0.16
0.12
0.04
Overall
T2DM
Obesity

Supplementary Figure S1. MASLD, MASL, and MASH prevalences across age subgroups in the overall population and in individuals with type 2 diabetes mellitus or obesity.
The graphs show the proportion of individuals with (**a**) MASLD, (**b**) MASL and (**c**) MASH across age subgroups.
MASLD, metabolic dysfunction-associated steatotic liver disease; MASL, metabolic dysfunction-associated steatotic liver; MASH, metabolic dysfunction–associated steatohepatitis; T2DM, type 2 diabetes mellitus.

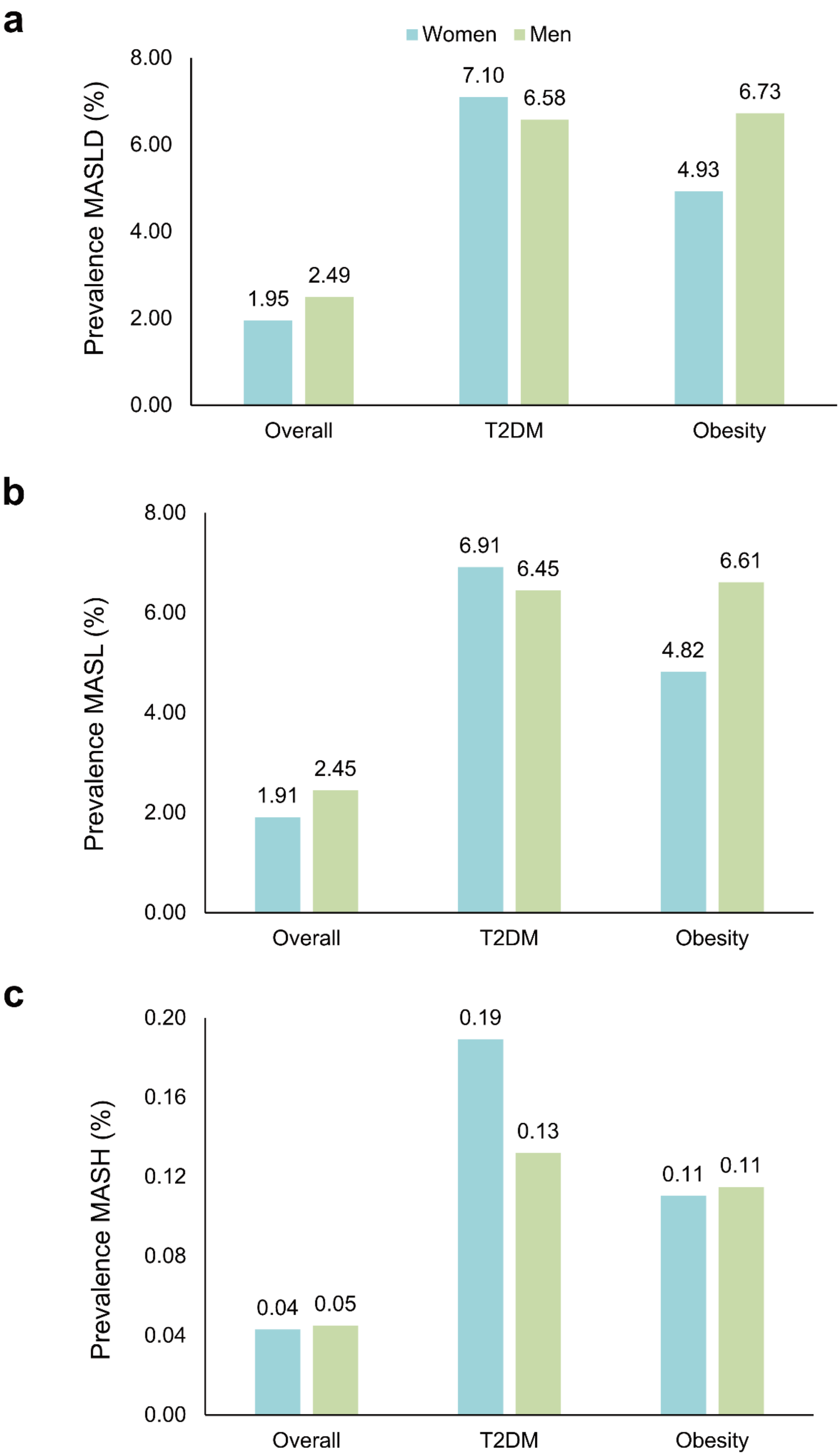
a
Women
Men
Prevalence MASLD (%)
8.00
6.00
4.00
2.00
0.00
1.95
2.49
7.10
6.58
4.93
6.73
Overall
T2DM
Obesity
b
Prevalence MASL (%)
8.00
6.00
4.00
2.00
0.00
1.91
2.45
6.91
6.45
4.82
6.61
Overall
T2DM
Obesity
c
Prevalence MASH (%)
0.20
0.16
0.12
0.08
0.04
0.00
0.04
0.05
0.19
0.13
0.11
0.11
Overall
T2DM
Obesity

Supplementary Figure S2. MASLD, MASL, and MASH prevalences across men and women in the overall population and in individuals with type 2 diabetes mellitus or obesity.
The graphs show the proportion of individuals with (**a**) MASLD, (**b**) MASL and (**c**) MASH across men and women. MASLD, metabolic dysfunction-associated steatotic liver disease; MASL, metabolic dysfunction-associated steatotic liver; MASH, metabolic dysfunction–associated steatohepatitis; T2DM, type 2 diabetes mellitus.
